# A U-shaped Association of Breastfeeding Duration with Cognitive Impairment in Chinese Postmenopausal Women

**DOI:** 10.1038/s41598-020-63599-z

**Published:** 2020-04-20

**Authors:** Fu-Dong Li, Jun-Fen Lin, Xu-Hua Ying, Yin-Wei Qiu, Song-Tao Li, Yu-Jia Zhai, Tao Zhang, Xin-Yi Wang, Xue Gu, Meng-Na Wu, Fan He

**Affiliations:** 1 Zhejiang Provincial Center for Disease Control and Prevention, Hangzhou, Zhejiang China; 2 Yuhuan Center for Disease Prevention and Control, Taizhou, Zhejiang China

**Keywords:** Geriatrics, Epidemiology, Risk factors

## Abstract

Breastfeeding is related to maternal health. However, the association of women’s breastfeeding duration with cognitive function in their later life is limited and inconsistent. The aim of this study was to accurately evaluate the association in Chinese postmenopausal women. We analyzed the data from Zhejiang Ageing and Health Cohort Study including 5487 postmenopausal women. Cognitive impairment was assessed via the Mini-Mental State Examination. Data on breastfeeding duration was collected in the reproductive history section within the questionnaire. Generalized additive models (GAMs) and logistic regression models, controlled for an extensive range of potential confounders, were generated to examine the associations. A U-shaped association was identified between breastfeeding duration and cognitive impairment based on GAM. The nadir with lowest odds of cognitive impairment was ascertained by quadratic model as 12 months. The logistic models showed that compared with women breastfeeding 12 months per child, the fully adjusted odds ratios (ORs) were 1.50 (95% Confidence Interval (CI): 1.20–1.88), 1.58 (95% CI: 1.29–1.93), 1.33 (95% CI: 1.06–1.68), 2.08 (95% CI: 1.64–2.65) for those averagely breastfeeding <6, 6-<12,>12–18,>18 months, respectively. Furthermore, we did not observe significant effect modification of the association. Future longitudinal studies are needed to confirm the association.

## Introduction

Cognitive impairment, a common symptom of dementia and its most common cause Alzheimer’s disease (AD), is becoming an important health issue in the elderly. Based on estimation, more than 35.6 million people lived with dementia worldwide in 2010; this number is likely to be doubled by 2030, and tripled by 2050^1^. The prevalence of dementia and AD in Chinese population aged 65 years and older were 5.14% and 3.21% respectively^2^, and the situation of cognitive impairment would be more serious.

Breastfeeding is recognized as the normal method for feeding infants worldwide^3^. It leads to various hormonal changes, and consequently affects the health of mothers. Several systematic reviews and meta-analyses demonstrated that breastfeeding reduced the risk of diseases in mothers, including breast cancer^4^, ovarian cancer^5^, type 2 diabetes mellitus^6^, hypertension^7^, etcetera. These studies provided evidence that breastfeeding had the capacity to influence disease risk by altering hormone profiles. Meanwhile, accumulating evidence showed that variation of sex hormones exposure in reproductive period may modify the risk of cognitive impairment and AD in women’s later life^8,9^. However, the epidemiological data on association between breastfeeding duration and risk of cognitive impairment or AD is limited and inconsistent. To our best knowledge, only two previous studies focused on this topic. Heys and colleagues^10^ reported that shorter average duration of breastfeeding per child was associated with better cognitive function. On the contrary, another study^11^ found that longer breastfeeding duration corresponded to lower AD risk. Both of the two studies tested the linear association regardless the possibility of curvilinear relationship, which may partially explain the inconsistency. Moreover, World Health Organization (WHO) recommends exclusive breastfeeding up to 6 months of age with continued breastfeeding up to 2 years or beyond^12^, which is not a short period. For maternal cognitive function, whether an appropriate or optimal duration of breastfeeding exists is worth further study.

Thus in this study, we utilized the cross-sectional data from a large population-based cohort study to investigate the linear or curvilinear association of breastfeeding duration with risk of cognitive impairment among Chinese postmenopausal women, and the possibility of optimal breastfeeding duration for cognitive function.

## Results

### Description of participants

By using participants selection criteria mentioned above, we initially identified 5635 postmenopausal women with at least one parity from an accumulation of 10801 participants. After checking for data completeness, we excluded women with missing data on cognitive impairment (n = 17) and covariates (n = 131). The final total of women included in the analysis was 5487. The general characteristics of included women are presented in Table 1.

**Table 1:** General characteristics of included participants.

General characteristics	Overall (N = 5487)	Cognitive impairment		P-value
		Yes (N = 1053)	No (N = 4434)	
Age (years, mean ± SD)	69.2 ± 7.7	73.0 ± 8.6	68.3 ± 7.2	<0.01
Race (N, %)				<0.01
Han	5326 (97.1)	1008 (95.7)	4318 (97.4)	
Minority	161 (2.9)	45 (4.3)	116 (2.6)	
Education level (N, %)				<0.01
<Primary	3454 (62.9)	742 (70.5)	2712 (61.2)	
Primary	1811 (33.0)	274 (26.0)	1537 (34.7)	
Junior middle	200 (3.6)	32 (3.0)	168 (3.8)	
≥Senior middle	22 (0.4)	5 (0.5)	17 (0.4)	
Marital status (N, %)				<0.01
Single	21 (0.4)	5 (0.5)	16 (0.4)	
Married	3638 (66.3)	540 (51.3)	3098 (69.9)	
Divorced/widowed	1828 (33.3)	508 (48.2)	1320 (29.8)	
Family income (N, %)				<0.01
≤10,000 CNY	715 (13.0)	231 (21.9)	484 (10.9)	
10,001~20,000 CNY	1120 (20.4)	244 (23.2)	876 (19.8)	
20,001~50,000 CNY	1860 (33.9)	288 (27.4)	1572 (35.5)	
50,001~100,000 CNY	1005 (18.3)	176 (16.7)	829 (18.7)	
>100,000 CNY	787 (14.3)	114 (10.8)	673 (15.2)	
Smoking (N, %)				0.43
Never	5433 (99.0)	1039 (98.7)	4394 (99.1)	
Past	14 (0.3)	4 (0.4)	10 (0.2)	
Current	40 (0.7)	10 (0.9)	30 (0.7)	
Alcohol drinking (N, %)				0.38
Never	4932 (89.9)	946 (89.8)	3986 (89.9)	
Past	99 (1.8)	24 (2.3)	75 (1.7)	
Current	456 (8.3)	83 (7.9)	373 (8.4)	
BMI (kg/m^2^, mean ± SD)	23.6 ± 3.4	23.4 ± 3.6	23.6 ± 3.4	0.02
Physical activity (yes, N, %)	1311 (23.9)	196 (18.6)	1115 (25.1)	<0.01
Hypertension (presence, N, %)	2586 (47.1)	543 (51.6)	2043 (46.1)	<0.01
Diabetes (presence, N, %)	632 (11.5)	121 (11.5)	511 (11.5)	0.98
CHD (presence, N, %)	190 (3.5)	45 (4.3)	145 (3.3)	0.11
ADL scores (mean ± SD)	99.3 ± 5.3	97.9 ± 10.1	99.6 ± 3.1	<0.01
PHQ-9 scores (mean ± SD)	1.7 ± 2.8	2.4 ± 3.5	1.6 ± 2.6	<0.01
MMSE scores (mean ± SD)	22.8 ± 5.6	13.9 ± 4.5	24.9 ± 3.2	<0.01

Abbreviations: SD, standard deviation; CNY, Chinese Yuan; BMI, body mass index; CHD, coronary heart disease; ADL, activities of daily living scale; PHQ-9, patient health questionnaire-9 scale.

Among the 5487 participants, 1053 were indicated with cognitive impairment by MMSE, and the prevalence of cognitive impairment was 19.2% (95% CI 18.2~20.2%). The mean MMSE score of participants was 22.8, and detail information on MMSE scores is presented in Table S1. The mean age of participants was 69.2 years, and participants with cognitive impairment was older than those with normal cognitive function (P < 0.01). Women with cognitive impairment had significantly higher proportions in subgroups of ethnic minority, lower education level, single/divorced/widowed, lower family income, no physical activity, and presence of hypertension. Furthermore, differences of BMI, ADL scores and PHQ-9 scores between women with and without cognitive impairment were statistically significant. In addition, general characteristics among women with different breastfeeding duration are presented in Table S2.

Descriptive statistics for reproductive characteristics of included women are shown in Table 2. The women in this study started menopause on average 32.5 years after menarche, with longer period in those without cognitive impairment. 93.8% of them reported regular menstrual cycle during reproductive period, and no significant difference was observed between groups with and without cognitive impairment. Younger age at first birth, higher parity, and less number of incomplete pregnancy were found in women with worse cognitive function. 200 (3.6%) women ever used oral contraceptive, with higher proportion among those without cognitive impairment. These reproductive characteristics would be further adjusted, except cycle regularity due to the insignificant result. Additionally, the distribution of mean breastfeeding duration was significantly different in those with and without cognitive impairment.

**Table 2:** Breastfeeding duration and other reproductive characteristics among included participants.

Reproductive characteristics	Overall (N = 5487)	Cognitive impairment		P value
		Yes (N = 1053)	No (N = 4434)	
Mean breastfeeding duration (N, %)				<0.01
<6 months	897 (16.3)	202 (19.2)	695 (15.7)	
6-<12 months	1399 (25.5)	295 (28.0)	1104 (24.9)	
12 months	1603 (29.2)	221 (21.0)	1382 (31.2)	
>12–18 months	895 (16.3)	167 (15.9)	728 (16.4)	
>18 months	693 (12.6)	168 (16.0)	525 (11.8)	
Reproductive period (years, mean ± SD)	32.5 ± 4.6	32.1 ± 4.6	32.6 ± 4.6	<0.01
Cycle regularity (regular, N, %)	5148 (93.8)	987 (93.7)	4161 (93.8)	0.89
Age at first birth (years, mean ± SD)	21.3 ± 2.5	21.1 ± 2.3	21.4 ± 2.5	<0.01
Parity (mean ± SD)	3.4 ± 1.6	4.0 ± 1.8	3.3 ± 1.5	<0.01
Incomplete pregnancy (mean ± SD)	0.5 ± 0.8	0.4 ± 0.8	0.5 ± 0.8	<0.01
Oral contraceptives use (ever, N, %)	200 (3.6)	26 (2.5)	174 (3.9)	0.02

Abbreviations: SD, standard deviation.

### Association between breastfeeding duration and cognitive impairment

**Figure 1:**
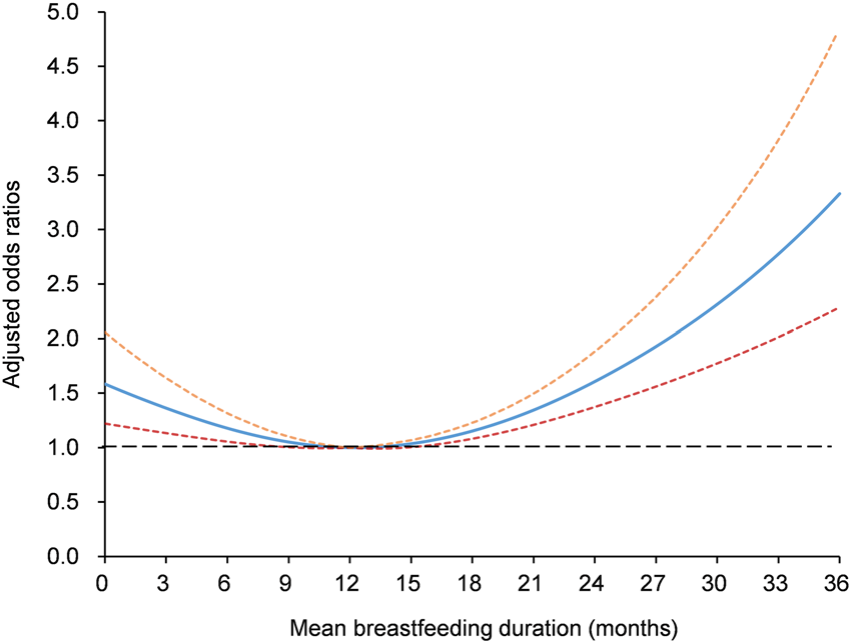
Smoothing plot for mean breastfeeding duration with cognitive impairment. The solid line indicates the point estimation for adjusted odds ratios (ORs) of cognitive impairment, and the dotted lines represent 95% confidence intervals (CIs).

The results of GAMs and quadratic models suggested a U-shaped association between mean breastfeeding duration and risk of cognitive impairment. Adjusted ORs for cognitive impairment using 12 months of mean breastfeeding duration as the reference demonstrated higher odds with longer as well as shorter mean breastfeeding duration (Fig. 1). Results of logistic regression models also indicated the U-shaped association, consistent with findings from GAMs. As shown in Table 3, compared with the reference group (12 months), the crude ORs (95% CIs) of cognitive impairment were 1.82 (1.47–2.25), 1.67 (1.38–2.02), 1.44 (1.15–1.79), and 2.00 (1.60–2.50) for <6 months, 6-<12 months, >12–18 months, and >18 months group, respectively. After adjusting for general characteristics in model 2, as well as further adjusting for other reproductive characteristics in model 3, the results showed no significant difference. The fully adjusted ORs (95% CIs) were 1.50 (1.20–1.88), 1.58 (1.29–1.93), 1.33 (1.06–1.68), and 2.08 (1.64–2.65) for women averagely breastfeeding <6, 6-<12, >12–18, >18 months, respectively.

**Table 3:** Association between mean breastfeeding duration and cognitive impairment.

Mean breastfeeding duration	Model 1^a^	P value	Model 2^b^	P value	Model 3^c^	P value
	OR (95% CI)		OR (95% CI)		OR (95% CI)	
<6 months	1.82 (1.47–2.25)	<0.01	1.54 (1.24–1.93)	<0.01	1.50 (1.20–1.88)	<0.01
6-<12 months	1.67 (1.38–2.02)	<0.01	1.63 (1.34–1.99)	<0.01	1.58 (1.29–1.93)	<0.01
12 months	1 (reference)	—	1 (reference)	—	1 (reference)	—
>12–18 months	1.44 (1.15–1.79)	<0.01	1.36 (1.08–1.71)	0.01	1.33 (1.06–1.68)	0.02
>18 months	2.00 (1.60–2.50)	<0.01	2.10 (1.66–2.67)	<0.01	2.08 (1.64–2.65)	<0.01

Abbreviations: OR, odds ratio; CI, confidence interval.

^a^No variable was adjusted in model 1.

^b^Adjusted for age, race, education level, marital status, family income, body mass index, physical activity, hypertension, Activities of Daily Living Scale scores, and patient health questionnaire-9 scores.

^c^Adjusted for same covariates in model 2 plus reproductive period, age at first birth, parity, incomplete pregnancy, and oral contraceptives use.

**Figure 2:**
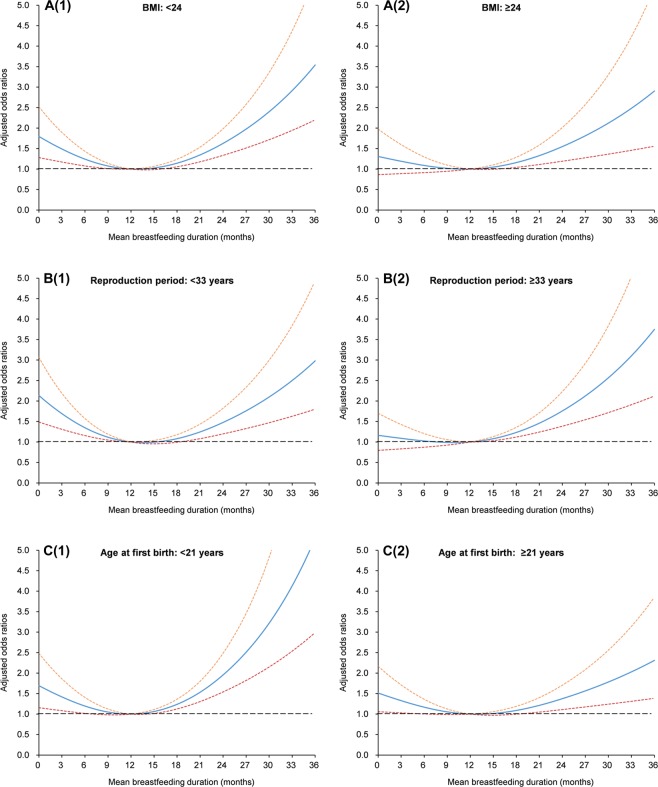
Smoothing plots for mean breastfeeding duration with cognitive impairment, stratified by BMI (**A**(**1**): <24 km/m^2^, **A**(**2**): ≥24 kg/m^2^), reproduction period (**B**(**1**): <33 years, **B**(**2**): ≥33 years), age at first birth (**C**(**1**): <21 years, **C**(**2**): ≥21 years), respectively. The solid line indicates the point estimation for adjusted odds ratios (ORs) of cognitive impairment, and the dotted lines represent 95% confidence intervals (CIs).

**Figure 3:**
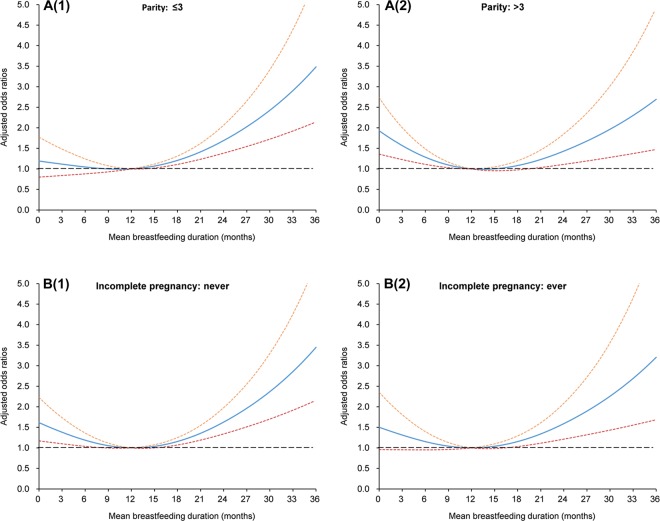
Smoothing plots for mean breastfeeding duration with cognitive impairment, stratified by parity (**A**(**1**): ≤3, **A**(**2**): >**3**), incomplete pregnancy (**B**(**1**): never, **B**(**2**): ≥ever), respectively. The solid line indicates the point estimation for adjusted odds ratios (ORs) of cognitive impairment, and the dotted lines represent 95% confidence intervals (CIs).

The results of both GAM and logistic models suggested that the U-shape association was relatively consistent across different subgroups stratified by BMI status and other reproductive characteristics (Figs. 2, 3 and Table 4). Meanwhile, no significant interaction was found between these variables and mean breastfeeding duration (Table 4).

**Table 4:** Adjusted ORs (95% CI) for cognitive impairment with mean breastfeeding duration, stratified by BMI status and other reproductive characteristics.

Subgroup	N	Mean breastfeeding duration				
		<6 months	6-<12 months	12 months	>12–18 months	>18 months
**BMI (kg/m**^**2**^**)**						
<24	3111	1.57 (1.18–2.10)	1.48 (1.13–1.93)	1 (reference)	1.35 (0.99–1.82)	1.94 (1.43–2.64)
≥24	2376	1.40 (0.98–2.00)	1.74 (1.27–2.37)	1 (reference)	1.33 (0.93–1.92)	2.30 (1.56–3.38)
P _Interaction_	0.59					
**Reproductive period (years)**						
<33	2532	1.70 (1.24–2.33)	1.92 (1.45–2.54)	1 (reference)	1.25 (0.90–1.73)	2.10 (1.51–2.94)
≥33	2955	1.31 (0.95–1.80)	1.31 (0.98–1.76)	1 (reference)	1.44 (1.03–2.01)	2.07 (1.47–2.92)
P _Interaction_	0.70					
**Age at first birth** **(years**)						
<21	2371	1.60 (1.14–2.24)	1.75 (1.30–2.37)	1 (reference)	1.36 (0.97–1.92)	2.57 (1.81–3.65)
≥21	3116	1.43 (1.06–1.93)	1.46 (1.11–1.91)	1 (reference)	1.32 (0.96–1.81)	1.72 (1.24–2.40)
P _Interaction_	0.26					
**Parity**						
≤3	3335	1.24 (0.89–1.73)	1.62 (1.22–2.15)	1 (reference)	1.40 (1.00–1.94)	2.09 (1.52–2.87)
>3	2152	1.75 (1.28–2.38)	1.56 (1.17–2.08)	1 (reference)	1.25 (0.90–1.74)	2.02 (1.40–2.92)
P _Interaction_	0.16					
**Incomplete pregnancy**						
Never	3464	1.61 (1.21–2.14)	1.77 (1.38–2.29)	1 (reference)	1.58 (1.18–2.11)	2.13 (1.58–2.86)
Ever	2023	1.33 (0.92–1.93)	1.23 (0.88–1.73)	1 (reference)	0.93 (0.62–1.40)	2.10 (1.40–3.15)
P _Interaction_	0.21					

Abbreviations: OR, odds ratio; CI, confidence interval; BMI, body mass index.

Adjusted for all same covariates included in model 3 (see Table 3), except the variable for stratification.

## Discussion

In this study, we explored the association of mean breastfeeding duration with cognitive impairment. The results of our study suggested a U-shaped association between mean breastfeeding duration and cognitive impairment. Mean breastfeeding duration of both longer and shorter than 12 months corresponded to poorer cognitive function in women’s later life. Furthermore, we did not observe significant effect modification of the association.

Our current findings are partially consistent with the previous population-based studies. One cohort study^11^ investigated the role of breastfeeding history on AD risk based on data from interviews of British older women. Ultimately 81 cases with sufficient information were included in analysis, suggesting that longer breastfeeding duration was associated with reduced AD risk. It should be noted that the maximum of mean breastfeeding duration was 12 months. The result of our study indicated that longer mean breastfeeding duration related with lower odds of cognitive impairment among women breastfeeding less than 12 months per child, consistent with that of British study. More importantly, our study possessed much larger sample size and explored the association not only among women breastfeeding less than 12 months per child, but also among those averagely breastfeeding more than 12 months. Another cross-sectional study^10^ of 11,094 Chinese older women found that shorter mean breastfeeding duration was associated with better cognitive function. Unfortunately, only age, education, parental possessions, occupation and physical activity were adjusted in this study, while our study adjusted much more potential confounders including other reproductive characteristics. That may partially explain the inconsistency. A recent population based study^13^ found no significant difference of the odds of cognitive impairment between Japanese women with and without breastfeeding history. However, the effect of breastfeeding duration on cognitive function was not explored in that study. The curvilinear association was demonstrated in our study using GAM and quadratic model, whose strengths included the ability to determine the natural shape of the nonlinear relationship, and to identify the optimal breastfeeding duration for cognitive function. Hence, our study might more accurately describe the association.

It remains unclear about the mechanisms responsible for the effects of breastfeeding duration on cognitive function. The endocrine change of lactation is complex. Longer breastfeeding duration usually suppresses ovulation longer and results in suppression of gonadotropins, which causes depressed production of plasma estradiol^14^. Animal and *in vitro* studies have identified that estrogen has several possible neuroprotective effects on the cognitive function^15^. Therefore, prolonged duration of breastfeeding-associated estrogen reduction may provide deleterious effects on cognitive health. Meanwhile, serum progesterone levels were generally low during lactation^16^. Progesterone has a heterogeneous effect on the brain, specifically in relation to cognitive function and AD. It was reported that independently progesterone exerted protective effects on cognitive function^17^. Nevertheless, some studies have found evidence that continuous progesterone inhibits estrogen protection from AD-related neuropathology^15^. Thus, the interactive effects of the two female sex steroid hormones are not quite straightforward, so do the effects of reduction in both of them during lactation. Oxytocin is released in a pulsatile manner during suckling^16^. It was demonstrated with positive effects on cognitive function^18^, which supported that breastfeeding accompanied with oxytocin release may contribute to lower risk of cognitive impairment. Prolactin is the key hormone of lactation^16^. Experimental studies discovered that prolactin was likely involved in the mediation of the rapid eye movement sleep (REMS)-promoting activity^19,20^, while REMS was associated with better cognitive function^21,22^. Consequently, prolactin release during lactation may lead to a benefit on cognition. In summary, the secretion of oxytocin and prolactin during breastfeeding may have beneficial effect on cognitive function, and estrogen deprivation contributes to detrimental effects, while progesterone presented beneficial or detrimental effects. The variation of different hormones exposure with their potential interactions may result in the non-linear relationship found in this study. Further research is warranted to elucidate the mechanism of breastfeeding’s effect on cognitive function.

Several methodological issues and limitations should be mentioned. First, the self-report of retrospective information on breastfeeding history may cause recall bias. One study found that under- and over-reporting occurred with equal frequency, and 55% of women were able to recall mean breastfeeding duration accurately within 1 month and 71% within 2 months^23^. Moreover, the association of breastfeeding duration with cognitive impairment might be affected if the error rate of recalling and summing over the breastfeeding duration of each child was higher among women with more severe forms of cognitive impairment. Second, considering the cross-sectional design, the reverse causation cannot be excluded on the occasion that women’s onset age of cognitive impairment was earlier than that of breastfeeding children. This situation may rarely occur because age is a major risk factor of cognitive impairment, which is not prevalent before the age of 65. Prospective studies are needed to ascertain the temporal relationship. Third, pregnancy interval, which was not concerned in the questionnaire, is also likely to influence the breastfeeding duration, with shorter interval between pregnancies resulting in shorter breastfeeding duration with the arrival of another child. Forth, we did not take genetic factors into consideration, such as apolipoprotein E (APOE)^24^ carrier status. Fifth, some factors which might probably affect the cognitive function were not measured in this study. Psychiatric history (e.g. schizophrenia) as well as other current psychiatric status (e.g. anxiety) may also result in cognitive decline. Meanwhile, we could not exclude the probability that cognitive impairment was caused by tumor, endocrine disorders, hormonal dysfunction, vitamin deficiency, vascular lesions, previous and anamnestic neurological disorders, etc. Participants did not undergo dementia protocol such as thyroid function, neurological tests and neuroimaging. Additionally, many of medication could have a negative effect on the cognitive performance. Finally, the results of our study should be interpreted cautiously. Although the lowest odds of cognitive impairment was found at 12 months of average breastfeeding, our finding does not mean 10 or 14 months of average breastfeeding is harmful, yet 12 months is protective. On one hand, 1 or 2 months error margin often exists when women recall breastfeeding duration, which was mentioned before. On the other hand, our results do support the protective effect of breastfeeding on maternal cognitive function, while appropriate duration was preferable (neither too short nor too long).

Despite these limitations, this study has a number of strengths. (1) With the application of GAMs and quadratic models which can more naturally describe the shape of curvilinear relationship, our study have originally identified the U-shaped association between breastfeeding duration and cognitive impairment. (2) Our study was based on a large community-based sample, which guaranteed the power of statistics. (3) A wide range of covariates including other aspects of reproductive characteristics were adjusted in multivariable analysis to minimize the potential confounding bias.

In conclusion, this study suggested a U-shaped association between breastfeeding duration and cognitive function among Chinese elderly women. Our findings are important, and have several implications for practice and cognitive impairment prevention. It supports the beneficial effects of breastfeeding for maternal health. Moreover, our results help mothers to decide the duration of breastfeeding children with regard to their cognitive function in later life. Future longitudinal studies are needed to confirm the association.

## Materials and Methods

### Study design

The data is from Zhejiang Ageing and Health Cohort Study^25^, a community based cohort study focusing on ageing and health among elderly in Zhejiang province, China. It was conducted by Zhejiang Provincial Center for Disease Control and Prevention. Seven counties were randomly selected from a total of 90 counties in Zhejiang province. One town in each county and several communities in each town were then randomly selected. All permanent residents aged ≥60 years old in these selected communities were expected to be included in the study. The baseline survey was finished at 2015, and 10911 elderly were recruited and completed the survey. One year later, the second round survey was finished in which 10801 elderly completed the survey (among 10911 baseline participants, 187 died and 9458 were successfully re-interviewed. In addition, 1343 was newly recruited).

A face to face interview based on a self-designed questionnaire was performed by trained research assistants for each participant both at baseline survey and second round survey. The questionnaire included the information of demographic characteristics, family status, reproductive history, medical disease, behavioral habits, diet habits, injury, depressive symptoms, self-care ability and cognitive function. Data were primarily checked by staff at Zhejiang Provincial Center for Disease Control and Prevention. Missing data and logical errors were fed back to the initial interviewer who would try to complete the dataset by reinvestigating the participants. In the questionnaire of second round survey, questions about breastfeeding duration were added into the reproductive history section, which were not involved in the questionnaire of baseline survey. Therefore, this study utilized the dataset of second round survey (n = 10801).

### Selection of participants

The study population was restricted to the postmenopausal women with at least one parity, which was selected from the dataset mentioned above. Female participants who answered “yes” to the question “Do you have menopause already?” and not “0” to the question “How many parity do you have?” were recognized as eligible participants. Data completeness was checked subsequently, and participants were excluded if they had any missing values on cognitive function and each reproductive factors.

### Assessment of cognitive function

Cognitive function was assessed using the Chinese version of the Mini-Mental State Examination (MMSE)^26^ within the questionnaire. The maximum score is 30 points, with higher MMSE scores indicating better cognitive function. The widely accepted cut-off score of cognitive impairment in China is education-specific^27^: 17/18 for illiteracy, 20/21 for people with primary education level, 24/25 for people with higher than primary education level. We defined cognitive impairment according to this standard.

### Breastfeeding duration

Data on breastfeeding duration was collected in the reproductive history section within the questionnaire. Our analysis focused on the mean breastfeeding duration, the ratio between total months spent on breastfeeding and number of full-term pregnancies. Total months spent on breastfeeding was determined from the response to the question “For how long do you breastfeed your children totally?” Number of full-term pregnancies was obtained from the response to the question “How many full-term pregnancies (live births) do you have?” In our analysis, mean breastfeeding duration were considered as both continuous variable and categorical variable. The method of categorizing will be described in ‘statistical analyses’ section.

### Covariates

Based on findings reported in the literature, general characteristics described below were considered as potential confounders and were included in our analysis: age (years, continuous variable), race (“Han ethnicity” and “minority”), education level (“lower than primary”, “primary”, “junior middle”, “senior middle” and “college and above”), marital status (“single”, “married” and “divorced/widowed”), family income (“≤10000”, “10001~20000”, “20001~50000”, “50001~100000” and “>100000” Chinese Yuan), smoking (“never”, “past” and “current”), alcohol drinking (“never”, “past” and “current”), body mass index (kg/m^2^, continuous variable), physical activity (“yes” and “no”), hypertension (“presence” and “absence”), diabetes (“presence” and “absence”), coronary heart disease (“presence” and “absence”), Activities of Daily Living Scale scores (continuous variable), Patient Health Questionnaire-9 scale (continuous variable). Moreover, following reproductive characteristics which might also affect the cognitive function were included for further adjustment: reproductive period (years, continuous variable), menstrual cycle regularity (“regular” and “irregular”), age at first birth (years, continuous variable), parity (continuous variable), incomplete pregnancies (continuous variable), and oral contraceptive use (“ever” and “never”). Detailed information on some of these variables were described as follows.

(1) Physical activity was assessed on the basis of the response (yes or no) to a single question about doing exercise regularly. (2) Medical disease section of the questionnaire contained the items on the presence or absence of 16 common diseases, which supposed to be formally diagnosed. Hypertension, diabetes and coronary heart disease (CHD) were considered in this study. (3) Body mass index (BMI) was calculated by dividing weight (kg) by the square of height (cm^2^). (4) The self-care ability was evaluated by the Barthel index of Activities of Daily Living Scale (ADL)^28^. It consists of self-feeding, bathing, dressing, toilet hygiene and functional mobility, with the total score of 100. (5) Depressive symptoms was evaluated using the Patient Health Questionnaire-9 scale (PHQ-9)^29^, nine-question version of the Primary Care Evaluation of Mental Disorders measured by self-reporting. Total score for the nine items ranges from 0 to 27, with greater values indicating increased severity. (6) Reproductive period, an indicator of endogenous estrogen exposure, was calculated as age at menopause minus age at menarche.

### Statistical analyses

Descriptive statistics were applied to illustrate the general characteristics and other reproductive characteristics of included postmenopausal women. The associations of general characteristics and reproductive characteristics with cognitive function were examined by t-test or Chi-square test or Kruskal-Wallis test as appropriate to variables. The variables which were associated with cognitive impairment at the 10% significance level (p < 0.1) were considered as potential confounders.

Generalized Additive Models (GAMs) were performed to explore the linear or curvilinear association between mean breastfeeding duration as continuous variable and risk of cognitive impairment, adjusted for all potential confounders. Restricted cubic spline smoothing technique^30^ was used for dependent variable (mean breastfeeding duration), and link function was Logit due to the binary outcome. Three knots were set at the 5th, 50th and 95th percentile^31^ according to the distributions of mean breastfeeding duration. As GAM analysis suggested a U-shaped association, quadratic regression was performed to determine the nadir where the risk is lowest: Pr (Cognitive impairment | mean breastfeeding duration) = exp [−α + β_1_ (mean breastfeeding duration) + β_2_ (mean breastfeeding duration)^2^], with parameters α, β_1_, and β_2_ estimated using maximum likelihood methods. If β_1_ and β_2_ are both nonzero and statistically significant, then the estimated level of mean breastfeeding duration at which risk of cognitive impairment is lowest equals −(β_1_/2β_2_). The value of nadir was used as reference for displaying the overall trend of ORs of cognitive impairment through the range of mean breastfeeding duration. Based on the quadratic regression, β_1_ and β_2_ were estimated as 0.049 and 0.002, respectively; both P values were less than 0.05. As a consequence, mean breastfeeding duration associated with lowest risk was identified as 12 months.

Meanwhile, this value (12 months) was used as a cut-off point for categorizing the mean breastfeeding duration. Individuals above and below the cut-off point were both stratified into 2 subgroups. Finally, individuals were divided into 5 groups: <6, 6-<12, 12, >12–18, >18 months. Then logistic regression models were performed to assess the risk of cognitive impairment with mean breastfeeding duration as categorical variable. Crude odds ratios (ORs), 95% confidence intervals (CIs) and corresponding P values of cognitive impairment associated with mean breastfeeding duration were calculated by binary logistic regression in model 1. Adjustment for general characteristics identified above was performed in model 2, then model 3 was generated by further including related reproductive characteristics.

Based on previous literature^8^, we suspected that BMI and other reproductive characteristics (reproductive period, age at first birth, parity, and incomplete pregnancy) might modify the effect of breastfeeding duration on risk of cognitive impairment. Menstrual cycle regularity and oral contraceptives use were not considered in this step due to the large difference of sample size between variable levels. The continuous variables among these factors were transformed into dichotomous variables for further stratification using the median as cut-off points. Subgroup analyses stratified on these factors were conducted for both continuous variable (in GAMs) and categorical variable (in logistic regression models) of mean breastfeeding duration. Furthermore, interactions between breastfeeding duration and these factors were checked through the addition of cross-product terms in the corresponding main-effects models. All statistical analyses were performed by SAS 9.3 (SAS Institute Inc., Cary, NC), and the significance level was at two-tailed probability <0.05.

### Ethical issues

The protocol was approved by the Ethics Committee of Zhejiang Provincial Center for Disease Control and Prevention, and written informed consents were obtained from all participants prior to the research. All methods were performed in accordance with the relevant guidelines and regulations.

## Supplementary information

Supplementary information.

## Data Availability

The data are not publicly available and the present authors cannot directly share the data, as the use of data requires the permission of the Zhejiang Provincial Center for Disease Control and Prevention.
