# Supplementary material for: A U-shaped Association of Breastfeeding Duration with Cognitive Impairment in Chinese Postmenopausal Women
Source: Sci Rep. 2020 Apr 20;10:6584. doi: 10.1038/s41598-020-63599-z (PMC7170904; doi:10.1038/s41598-020-63599-z)
Supplement: Supplementary file 1 — Supplementary information. [file 41598_2020_63599_MOESM1_ESM.pdf]

Title of the manuscript: A U-shaped Association of Breastfeeding Duration with Cognitive Impairment in Chinese Postmenopausal Women

Author list: Fu-Dong Li, Jun-Fen Lin, Xu-Hua Ying, Yin-Wei Qiu, Song-Tao Li, Yu-Jia Zhai, Tao Zhang, Xin-Yi Wang, Xue Gu, Meng-Na Wu, Fan He*

* Corresponding author

Table S1. MMSE scores for each item of the included participants

Item	Subtotal score of the item	Overall (N=5487)	Cognitive impairment	
			Yes (N=1053)	No (N=4434)
Orientation-date	5	4.18 ± 1.27	2.54 ± 1.51	4.57 ± 0.82
Orientation-place	5	4.50 ± 1.02	3.29 ± 1.49	4.79 ± 0.58
Registration	3	2.66 ± 0.73	1.80 ± 1.06	2.87 ± 0.40
Attention & calculation	5	3.45 ± 1.81	1.32 ± 1.37	3.96 ± 1.50
Recall	3	2.21 ± 1.04	1.00 ± 0.99	2.50 ± 0.83
Naming	2	1.93 ± 0.30	1.76 ± 0.55	1.97 ± 0.18
Repetition	1	0.89 ± 0.31	0.63 ± 0.48	0.96 ± 0.20
3-Stage command	3	2.43 ± 0.94	1.42 ± 1.14	2.67 ± 0.70
Reading	1	0.31 ± 0.46	0.08 ± 0.28	0.37 ± 0.48
Writing	1	0.12 ± 0.32	0.02 ± 0.13	0.14 ± 0.35
Copy design	1	0.13 ± 0.34	0.02 ± 0.16	0.16 ± 0.37

All results were presented as mean ± standard deviation

Table S2. General characteristics among participants with different mean breastfeeding duration

General characteristics	Overall	Mean breastfeeding duration (months)					P-value
		<6	6-<12	12	>12-18	>18	
Age (years, mean ±SD)	69.16 ±7.72	71.51 ±7.51	69.10 ±7.60	68.37 ±7.70	68.94 ±7.69	68.36 ±7.72	<0.01
Race (N, %)							<0.01
Han	5326 (97.07)	880 (98.10)	1370 (97.93)	1584 (98.81)	861 (96.20)	631 (91.05)	
Minority	161 (2.93)	17 (1.90)	29 (2.07)	19 (1.19)	34 (3.80)	62 (8.95)	
Education level (N, %)							<0.01
<Primary	3454 (62.95)	589 (65.66)	883 (63.12)	931 (58.08)	613 (68.49)	438 (63.20)	
Primary	1811 (33.01)	282 (31.44)	459 (32.81)	569 (35.50)	262 (29.27)	239 (34.49)	
Junior middle	200 (3.64)	21 (2.34)	51 (3.65)	94 (5.86)	19 (2.12)	15 (2.16)	
≥Senior middle	22 (0.40)	5 (0.56)	6 (0.43)	9 (0.56)	1 (0.11)	1 (0.14)	
Marital status (N, %)							0.02
Single	21 (0.38)	3 (0.33)	2 (0.14)	6 (0.37)	5 (0.56)	5 (0.72)	
Married	3638 (66.30)	567 (63.21)	919 (65.69)	1111 (69.31)	575 (64.25)	466 (67.24)	
Divorced/widowed	1828 (33.32)	486 (30.32)	327 (36.45)	478 (34.17)	315 (35.20)	222 (32.03)	
Family income (N, %)							<0.01
≤10,000 CNY	715 (13.03)	121 (13.49)	204 (14.58)	197 (12.29)	114 (12.74)	79 (11.40)	
10,001~20,000 CNY	1120 (20.41)	203 (22.63)	287 (20.51)	350 (21.83)	149 (16.65)	131 (18.90)	
20,001~50,000 CNY	1860 (33.90)	261 (29.10)	441 (31.52)	670 (41.80)	279 (31.17)	209 (30.16)	

50,001~100,000 CNY	1005 (18.32)	178 (19.84)	268 (19.16)	235 (14.66)	170 (18.99)	154 (22.22)	
>100,000 CNY	787 (14.34)	134 (14.94)	199 (14.22)	151 (9.42)	183 (20.45)	120 (17.32)	
Smoking (N, %)							0.07
Never	5433 (99.02)	881 (98.22)	1389 (99.29)	1595 (99.50)	884 (98.77)	684 (98.70)	
Past	14 (0.26)	3 (0.33)	4 (0.29)	2 (0.12)	2 (0.22)	3 (0.43)	
Current	40 (0.73)	13 (1.45)	6 (0.43)	6 (0.37)	9 (1.01)	6 (0.87)	
Alcohol drinking (N, %)							<0.01
Never	4932 (89.89)	804 (89.63)	1274 (91.07)	1462 (91.20)	814 (90.95)	578 (83.41)	
Past	99 (1.80)	14 (1.56)	17 (1.22)	24 (1.50)	12 (1.34)	32 (4.62)	
Current	456 (8.31)	79 (8.81)	108 (7.72)	117 (7.30)	69 (7.71)	83 (11.98)	
BMI (kg/m^2, mean ± SD)	23.57 ± 3.44	23.41 ± 3.47	23.63 ± 3.35	23.77 ± 3.47	23.69 ± 3.44	23.02 ± 3.42	<0.01
Physical activity (yes, N, %)	1311 (23.89)	201 (22.41)	349 (24.95)	417 (26.01)	186 (20.78)	158 (22.80)	0.03
Hypertension (presence, N, %)	2586 (47.13)	495 (55.18)	662 (47.32)	732 (45.66)	419 (46.82)	278 (40.12)	<0.01
Diabetes (presence, N, %)	632 (11.52)	102 (11.37)	159 (11.37)	194 (12.10)	116 (12.96)	61 (8.80)	0.11
CHD (presence, N, %)	190 (3.46)	44 (4.91)	47 (3.36)	50 (3.12)	25 (2.79)	24 (3.46)	0.12
ADL scores (mean ± SD)	99.26 ± 5.27	98.86 ± 6.44	99.22 ± 5.95	99.44 ± 4.33	99.36 ± 5	99.29 ± 4.35	<0.01
PHQ-9 scores (mean ± SD)	1.73 ± 2.85	1.47 ± 2.69	1.66 ± 2.86	1.63 ± 2.73	2.3 ± 3.27	1.74 ± 2.6	<0.01
